# Seroprevalence of Epizootic Hemorrhagic Disease Virus in Cattle, Sheep, and Goats in New York State From 2022 Through 2025

**DOI:** 10.1155/tbed/5552390

**Published:** 2026-09-28

**Authors:** Elisha Frye, Patrick Connelly, Krysten Schuler, Jennifer Powers, Melissa Aprea, Diego G. Diel

**Affiliations:** ^1^ Department of Population Medicine and Diagnostic Sciences, College of Veterinary Medicine, Cornell University, Ithaca 14853, New York, USA, cornell.edu; ^2^ Animal Health Diagnostic Center, College of Veterinary Medicine, Cornell University, 240 Farrier Road, Ithaca 14853, New York, USA, cornell.edu; ^3^ Louis Calder Center Biological Field Station, Fordham University, 31 Whippoorwill Rd, Armonk 10504, New York, USA, fordham.edu; ^4^ Department of Public and Ecosystem Health, Cornell University College of Veterinary Medicine, Ithaca 14853, New York, USA

**Keywords:** antibodies, cattle, epizootic hemorrhagic disease, goat, New York State, seroprevalence, sheep, white-tailed deer

## Abstract

Epizootic hemorrhagic disease (EHD) virus (EHDV) is a vector‐borne virus transmitted by *Culicoides* midges that infects domestic and wild ruminants. EHDV causes high mortality in white‐tailed deer (WTD) and oral lesions mimicking foot and mouth disease (FMD) in cattle. Small ruminants (e.g., domestic sheep, domestic goats) seroconvert but do not typically develop disease. In 2021, ~2100 WTD deaths were attributed to EHD in New York (NY) State. That same year, an EHD outbreak at a NY dairy resulted in a foreign animal disease (FAD) investigation to rule out FMD, and EHDV was detected in a captive bison herd in NY. In response to the EHD outbreak affecting wildlife, agricultural, and exhibition animals, serum samples from cattle, sheep, and goats submitted to the Cornell University College of Veterinary Medicine Animal Health Diagnostic Center (AHDC) were tested for EHDV antibodies. The samples tested in our surveillance study included samples collected during and after *Culicoides* vector activity (July–January) over three seasons from 2022 to 2025. A total of 5698 samples comprising 4874 cattle and 824 small ruminants were tested using the agar gel immunodiffusion assay (AGID). Season 1 had 0.6% seroprevalence (10/1719), season 2 had 0.7% seroprevalence (15/2021), season 3 had 0.5% seroprevalence (9/1958) with an overall seroprevalence of 0.6%. Neutralizing antibody (NA) titers were determined on all AGID positive samples for EHDV serotypes 1, 2, and 6. There were 20 samples positive for EHDV serotype 2 (NA titers from 1:40 to ≥ 1:1280); 11 samples positive for EHDV serotype 6 (NA titers from 1:10 to 1:640); and three animals with cross‐reaction amongst serotypes. Of the 34 positive samples, 31 were from cattle and 3 were from sheep. Nineteen samples were from counties with large numbers of WTD deaths attributed to EHDV in 2021 or counties with cases of EHDV in cattle. This data indicates that EHDV serotypes 2 and 6 subclinically infected a small number of domestic ruminants in NY from 2022 to 2025.

## 1. Introduction

Epizootic hemorrhagic disease (EHD) virus (EHDV) is a vector‐borne virus that infects both domestic and wild ruminants, classified in the family Sedoreoviridae, genus *Orbivirus* It is a nonenveloped, double‐stranded RNA virus with an icosahedral capsid and three layers; the outer capsid, the inner, and core layers. The outer capsid consists of two structural proteins, VP2 and VP5. The inner layer consists of VP3 and VP7, and the core includes VP1, VP4, and VP6. Nonstructural proteins include NS1, NS2, NS3/NS3A, and NS4 [1]. EHDV is closely related to bluetongue virus (BTV) [2–4]. As early as the 1800s, EHDV was anecdotally reported by deer hunters as “black tongue” and was first identified in the United States (US) in New Jersey in August of 1955, when an estimated 500–700 white‐tailed deer (WTD) died [5]. Dead deer were found near water sources, with evidence of edema and internal hemorrhage on postmortem examination [6]. To date, seven EHDV serotypes have been identified and designated EHDV 1–2 and 4–8, including a strain of EHDV‐2 called *Ibaraki* virus, with other serotypes still being discovered, including EHDV‐10 and an unnamed serotype [1, 6–8]. In the US, serotypes 1, 2, and 6 have been detected [9–11]. *Culicoides* biting midges transmit the virus [11, 12] and are most abundant in the summer [13], resulting in a seasonal pattern of disease limited to the warmer months [11]. In North America, EHDV is transmitted by *Culicoides sonorensis* and *Culicoides insignis* [14], but other species of *Culicoides* may also transmit the virus [3, 13, 15, 16]. Periods of drought, which can create productive midge breeding habitats due to shallow water and exposed mud, have correlated with increased EHDV mortality in WTD [10, 11, 17, 18]. EHDV is in the National List of Reportable Animal Diseases [19] and occurs as an endemic disease in the Southern US [16] but as an epidemic disease in the Northern US [17]. New York (NY) represents an incursive zone, where outbreaks are very infrequent but can be severe due to lack of herd immunity in susceptible populations like WTD [11, 17].

EHDV may cause high morbidity and mortality in captive and wild WTD [3, 5] and has been documented to cause morbidity in other species of deer, cattle, bison, and yaks [4, 10, 11, 18, 20]. Sheep and goats develop antibodies to EHDV but rarely exhibit signs of clinical disease [4, 11, 20]. Cattle and bison typically have inapparent infections but may develop mild disease and present with oral vesicular lesions, drooling, inappetence, fever, lameness, and in the case of *Ibaraki* virus, abortions, stillbirths, and decreased milk production [4, 18, 21, 22]. The oral lesions make EHDV a differential diagnosis for foot and mouth disease (FMD), a foreign animal disease (FAD) in the US [2, 18, 19]. Thus, detection of oral lesions in cloven‐hoofed animals results in a FAD investigation as FMD must be ruled out. In the fall of 2012, there was an extensive EHD outbreak in captive and wild WTD, cattle, and bison in Ohio, Michigan, Missouri, Iowa, Nebraska, and South Dakota, with both serotypes 2 and 6 detected [18]. Most cattle had salivation, oral lesions, and decreased appetite. The morbidity rate was 7%, but decreased milk production made this a costly outbreak for producers. A total of 83 FAD investigations were performed by veterinary regulatory officials (80 bovine and 3 bison cases) [18].

EHDV was initially found in WTD in NY in 2007 and detected again in 2011 [23]. These outbreaks were caused by serotypes 1 and 2. Serotype 6 has been detected in the US since 2006 [17], and all three serotypes are known to be circulating in ruminant populations. The virus was not detected frequently in NY until 2020 and 2021, when an estimated 1500 and 2100 WTD mortalities were attributed to EHDV, respectively, caused by serotypes 2 and 6 [23, 24]. Most deaths were concentrated in the Hudson Valley, eastern Lake Ontario drainage basin, and Long Island regions of NY [23–25].

Sporadic EHDV prevalence studies have been performed on cattle, bison, and captive deer over the last few decades in Texas [26], the Southeast [27, 28], the West, and the Midwest [21, 29, 30], but none covered the Northeast. NY is the fifth largest dairy state in the US, with 630,000 dairy cattle and 2864 dairy farmers [31]. The dairy sector is a major component of the NY agricultural economy. Given that cattle can be clinically affected, which impacts productivity, or subclinical, with prolonged viremia up to 50 days [10, 11], cattle have the potential to serve as an EHDV reservoir for WTD.

Here, we describe clinical and diagnostic findings of EHD in domestic ruminants during the concomitant outbreak in WTD. These cases spurred an investigation into seroprevalence and serotypes of EHDV among cattle mainly, but also sheep and goats, over three seasons (July 2022–January 2025) across NY state, with a focus on samples from regions with reported WTD mortality due to EHD and regions with high concentrations of dairy farms.

## 2. Materials and Methods

### 2.1. Clinical Domestic Animal Cases

In October 2021, a dairy herd in Suffolk County on Long Island reported four cattle presenting with drooling, anorexia, and lameness. An FAD investigation was initiated; samples of whole blood were submitted to the USDA–National Veterinary Services Laboratories (NVSL) and tested positive for EHDV. Two other cases of EHD were diagnosed in beef cattle in Vermont (VT) along the NY border in the fall of 2021; 1 of the cases was diagnosed at the Cornell University College of Veterinary Medicine Animal Health Diagnostic Center (AHDC) by real‐time RT‐PCR using a previously published method that amplifies the NS1 gene, which is conserved across EHDV serotypes 1–8 [32]. Also in October 2021, three bison from a captive bison herd in NY had facial and ocular swelling, conjunctivitis, and bloody nasal discharge. The index case was sequenced as EHDV‐2. The full genome of EHDV‐2 was obtained in the Virology Laboratory at the AHDC using validated sequence‐independent, single‐primer amplification and the Oxford Nanopore MinION sequencing platform. Sequences were deposited in GenBank under Accession Numbers PZ840468–PZ840477. Two other bison had robust EHDV‐2 neutralizing antibody (NA) titers detected in the serum in February 2022. The index case was real‐time RT‐PCR positive for EHDV with a moderate viral load (copy threshold [Ct] 28.85) in whole blood 5 months after the initial infection in March 2022. There were no clinical cases of EHD during the vector season (July through September) of 2022 in cattle in NY. In September 2023, there were three cows with oral vesicular lesions on a small dairy farm in Montgomery County, NY. After ruling out FMD, all 3 were EHDV real‐time RT‐PCR positive and confirmed as EHDV‐2 by partial genetic sequencing (GenBank Accession Numbers PZ840478–PZ840507). In October 2024, a single 8‐year‐old beef cow in a small 15‐cow herd in Washington county, NY, had necrotic ulcerated lesions on the soft palate; it was real‐time RT‐PCR positive for EHDV and confirmed as EHDV‐2 by partial genetic sequencing (GenBank Accession Numbers PZ840508–PZ840510).

### 2.2. Serology Sample Set and Testing

The AHDC receives serum samples from domestic ruminants from NY state for a variety of diagnostic assays. We extracted data from the laboratory information management system (LIMS) on a weekly basis for the following periods: from July 2022 to January 2023, from July 2023 to January 2024, and from July 2024 to January 2025. These periods were selected to capture samples collected during and after the *Culicoides* vector season in NY to maximize the likelihood of detecting EHDV antibodies. Serum submitted for bovine viral diarrhea virus antigen capture enzyme‐linked immunosorbent assay (ELISA) (BVDV PIX2 Test, Idexx Laboratories, Inc. catalog # 99‐20589), with the assumption that these animals were generally healthy and being screened for persistent infection (PI) status, was used. A similar data extraction was conducted monthly during the collection period for sheep and goat serum tested for small ruminant lentivirus antibodies by cELISA (Small Ruminant Lentivirus Antibody Test Kit, cELISA, Veterinary Medical Research and Development [VMRD] catalog # 289‐2). Submission forms were manually reviewed, and samples from animals younger than 3–4 months of age were omitted, when possible, to avoid potential interference from maternally derived antibodies [33]. In some instances, animals were designated as “adult,” and the precise age was unavailable. The county of origin was used to target samples from regions with documented EHD‐associated mortality events in WTD based on reports from the Cornell Wildlife Health Laboratory [24] and from high‐density dairy farming areas. If the farm’s county of origin was not provided (a rare occurrence), the county of the submitting veterinarian was used as a proxy.

The selected serum samples were stored frozen at −20°C within 1 month of receipt at the AHDC. For seasons 1 and 2, serum was batched and tested for the presence of EHDV antibodies by agar gel immunodiffusion assay (AGID) using reagents obtained from the USDA–NVSL. Approximately 15 mL of agar was dispensed into 100 mm × 15 mm petri plates and allowed to solidify. Using a template, seven patterns of seven wells each were cut into the agar. Each pattern consisted of six peripheral wells arranged in a circle surrounding a central well. The agar plugs were removed, and undiluted known antigen, known antibody, positive and negative controls, and test samples were added to the designated wells. The plates were incubated at room temperature for 24 h and then examined for the presence of precipitin lines. If the samples were positive on the EHDV AGID, virus neutralization for EHDV‐1, 2, and 6 was performed. Test serum samples were diluted 1:5 in minimum essential medium (MEM) with Earle’s salts supplemented with 2% penicillin–streptomycin. Two‐fold serial dilutions of each test serum were prepared in 96‐well microtiter plates, ranging from 1:5 to 1:640. A target virus concentration of 100 TCID_50_ per 50 µL was added to each serum dilution, resulting in final serum dilutions ranging from 1:10 to 1:1280. The serum‐virus mixtures were incubated for ~1 h at 37°C. Following incubation, baby hamster kidney‐21 (BHK‐21) indicator cells were added to each well. The plates were incubated for 4 days at 37°C in a humidified atmosphere containing 5% CO_2_ and subsequently examined microscopically for the presence of virus‐induced cytopathic effect (CPE). The NA titer was defined as the reciprocal of the highest serum dilution at which less than a 25% CPE was observed. The method was performed in accordance with the recommendations described in the WOAH Terrestrial Manual, Chapter 3.1.7, Section 2. If virus neutralization was negative for all three serotypes, we performed BTV cELISA (BTV Antibody Test Kit, cELISA v2, VMRD catalog # 5010.20‐2), as there is known cross reactivity between EHDV and BTV antibodies on the AGID test. For season 3, these reagents were not available, so the BTV AGID kit (VMRD cat# 288‐100) (https://www.vmrd.com/test-kits/bluetongue-virus-antibody-test-kit%2C-agid) was used for the initial screening. The kit insert states that “Antibodies to epizootic hemorrhagic disease virus are also detected” [34]. If the sample was positive on the BTV AGID, virus neutralization for EHDV‐1, 2, and 6 was performed. In instances where the virus neutralization was negative for all three serotypes, BTV cELISA was performed for confirmation. To ensure cross‐reaction between the EHDV AGID seropositive results from seasons 1 and 2 and the BTV AGID kit used in season 3, we tested all positive samples from seasons 1 and 2 with the BTV AGID, except for four original samples that had an insufficient quantity of serum. Of the 23 available EHDV AGID positive samples, 20 were positive, 2 were inconclusive, and 1 was negative on the BTV AGID. This shows 87% agreement between the assays in this small sample set. All serological testing was performed in the Virology Laboratory at the AHDC, and the assays described above are routinely used for serological testing for EHDV in clinical samples.

## 3. Results

In season 1, 1719 serum samples, consisting of 1370 bovine, 194 caprine, and 155 ovine, were tested. Ten samples were positive: nine bovine and one ovine (Figure 1A), resulting in a seroprevalence of 0.6%. Table 1 describes the signalment, county of origin, and virus neutralization endpoint for EHDV‐seropositive animals in the data set. All virus neutralization endpoints are listed, but when samples had a single‐serotype high virus neutralization result and low levels of cross‐reaction to the other serotypes, the result was categorized as positive for the highest serotype. Six cattle were positive for EHDV‐2: four cattle from Oswego County, with NA titers ranging from 1:40 to 1:160, and one cow each from Colombia and Washington Counties with titers of 1:320 and 1:80, respectively. One sample from Oswego County was accidentally included in testing as it was a 3‐month‐old Jersey calf and did not fit the study criteria for inclusion. It should be noted that this could have been the detection of maternal antibodies [33]. Three cattle were positive for EHDV‐6: two from Montgomery County with titers of 1:640 and 1:160 and one1 from Dutchess County with a titer of 1:160. The single seropositive sheep was in Ulster County and had a titer of 1:40 for serotypes 1 and 6, indicating a cross‐reaction between the two serotypes. All positive samples were submitted July–September 2022 (Figure 2).

**Figure 1:**
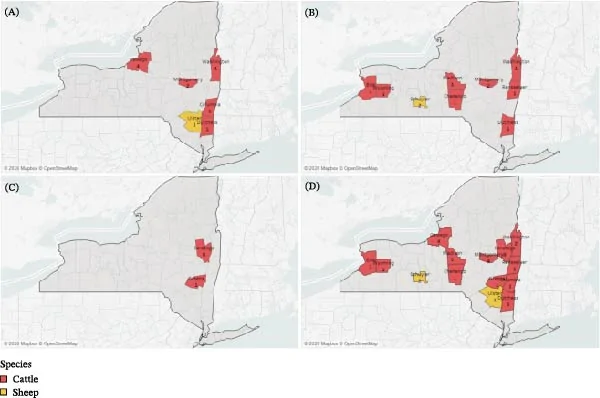
Epizootic hemorrhagic disease virus (EHDV) seropositive samples in New York State by county for cattle, sheep, and goats during seasons 1, 2, and 3 (A–C), and total EHDV seropositive samples for all three seasons (D).

**Figure 2:**
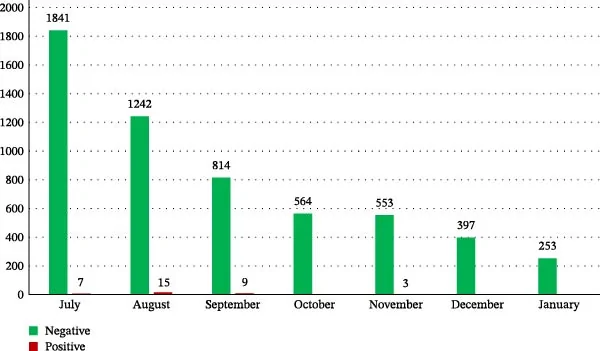
Epizootic hemorrhagic disease virus seronegative and seropositive samples by month of total study (July through January for seasons 1, 2, and 3).

**Table 1:** Description of epizootic hemorrhagic disease virus (EHDV) seropositive sample during the study period (three vector seasons from July 2022 through January 2025), including date of sample collection, animal signalment, county of origin, and virus neutralizing antibody (NA) endpoint for EHDV serotypes 1, 2, and 6.

Month/year	Species, breed	Age	Sex	County	EHD type 1 NA	EHD type 2 NA	EHD type 6 NA
7/2022	Ovine, Icelandic	Adult	Female	Ulster^a^	1:40	Negative	1:40
8/2022	Bovine, Hereford	19 months	Female	Oswego^a^	1:40	1:160	1:20
8/2022	Bovine, Jersey	3 months	Female	Oswego^a^	1:20	1:40	1:20
8/2022	Bovine, Ayrshire	2.5 years	Female	Oswego^a^	1:80	1:160	1:10
8/2022	Bovine, Shorthorn	17 months	Female	Oswego^a^	Negative	1:160	1:10
8/2022	Bovine, Mini Hereford	6 years	Female	Columbia^ab^	1:40	1:320	1:40
8/2022	Bovine, Guernsey	8 years	Female	Washington^c^	1:20	1:80	Negative
8/2022	Bovine, Angus cross	1 year	Male	Montgomery^c^	1:80	1:20	1:640
8/2022	Bovine, Black Baldy	1 year	Male	Montgomery^c^	1:40	1:10	1:160
9/2022	Bovine, Jersey	12 year	Female	Dutchess^a^	1:80	1:80	1:160
7/2023	Bovine, Angus	16 months	Female	Montgomery^c^	1:20	1:320	1:80
7/2023	Bovine, Holstein	5 year	Female	Madison	Negative	1:640	1:80
7/2023	Bovine, Holstein	3 year	Female	Erie	1:10	1:160	1:80
7/2023	Bovine, Polled Hereford	6 months	Female	Washington^c^	Negative	1:160	1:40
7/2023	Bovine, mixed breed	16 months	Female	Wyoming	1:40	≥1:1280	1:320
8/2023	Bovine, Jersey	15 months	Female	Chenango	Negative	Negative	1:10
8/2023	Bovine, Holstein^d^	3 years	Female	Dutchess^a^	1:10	1:80	1:320
8/2023	Bovine, Guernsey^d^	2 years	Female	Dutchess^a^	Negative	1:40	1:640
8/2023	Bovine, Holstein^d^	2 years	Female	Dutchess^a^	Negative	1:40	1:320
8/2023	Bovine, mixed breed^d^	2 years	Female	Dutchess^a^	1:20	1:80	1:640
8/2023	Bovine, Holstein^d^	22 months	Female	Dutchess^a^	1:10	1:20	1:320
9/2023	Bovine, Angus	Adult	Female	Rensselaer	1:10	1:10	1:160
11/2023	Bovine, Hereford	12 years	Female	Montgomery	1:20	1:80	1:20
11/2023	Ovine, Katahdin^d^	18 months	Female	Schuyler	Negative	1:40	1:20
11/2023	Ovine, Katahdin^d^	1 year	Female	Schuyler	Negative	1:10	1:10
8/2024	Bovine, White Park	9 years	Female	Greene^a^	1:10	Negative	1:160
7/2024	Bovine, Holstein	3 years	Female	Saratoga	1:10	1:80	1:40
9/2024	Bovine, Holstein^d^	2 years	Female	Saratoga	1:40	1:160	1:80
9/2024	Bovine, Holstein^d^	2 years	Female	Saratoga	1:20	1:160	1:80
9/2024	Bovine, Holstein^d^	2 years	Female	Saratoga	1:10	1:80	1:80
9/2024	Bovine, Holstein^d^	2 years	Female	Saratoga	1:10	1:160	1:40
9/2024	Bovine, Holstein^d^	2 years	Female	Saratoga	1:10	1:320	1:160
9/2024	Bovine, Holstein^d^	3 years	Female	Saratoga	1:10	1:320	1:40
9/2024	Bovine, Holstein^d^	2 years	Female	Saratoga	Negative	1:80	1:40

^a^Indicates a county with >50–500 white‐tailed deer deaths attributed to EHDV in 2021.

^b^The submitting veterinarian county is listed because owner county was not available in submission form.

^c^Indicates counties that had EHD clinical cases in cattle.

^d^Indicates animals within that cell were from the same premise and sampled on the same date.

In season 2, a total of 2021 serum samples, consisting of 1735 bovine, 243 caprine, and 43 ovine, were tested. Fifteen samples were positive: 13 bovine and 2 ovine (Figure 1B), resulting in a seroprevalence of 0.7%. Six cattle were positive for EHDV‐2: two from Montgomery County with NA titers of 1:320 and 1:80, and one bovine each from Erie (titer 1:160), Madison (titer 1:640), Washington (titer 1:160), and Wyoming (titer ≥ 1:1280) Counties. Seven cattle were positive for EHDV‐6: five from the same premise in Dutchess County with NA titers of 1:320 and 1:640 and one each from Chenango (titer 1:10) and Rensselaer (titer 1:160) Counties. Two sheep on the same premise were EHDV positive in Schuyler County, with one sheep positive for EHDV‐2 with a NA titer of 1:40 and the other sheep low positive with a titer of 1:10 for EHDV‐2 and 6 (Table 1). Positive samples were mostly submitted in July and August 2023, but two sheep in Schuyler County and one cow in Montgomery County were submitted in November 2023 (Figure 2).

In season 3, a total of 1958 serum samples, consisting of 1769 bovine samples (one sample was from a yak but was grouped with the bovine samples), 150 caprine samples, and 39 ovine samples (1 sample was from a big‐horn sheep from a zoo), were tested. Nine bovine samples were positive (Figure 1C), resulting in a seroprevalence of 0.5%. Eight cattle were positive for EHDV‐2, all from Saratoga County, with NA titers of 1:80, 1:160, and 1:320. Seven of these cattle were from the same premise, and one additional cow from this premise had a NA titer of 1:80 for EHDV‐2 and 6. One cow was positive for EHDV‐6 from Greene County with a titer of 1:160 (Table 1). EHDV‐positive samples were submitted July–September 2024 (Figure 2).

Overall, 4874 bovine serum samples and 824 small ruminant samples from NY were tested over three consecutive vector seasons from July 2022 to January 2025, for a total of 5698 samples (Figure 3). The seroprevalence for the study period was 0.6%, with 34 seropositive results (Figure 1D). In Table 2, the total number of samples tested per NY county, by month, is listed from July through January, for 2022–2025. Counties with >50–500 WTD deaths attributed to EHD in 2021 are indicated, along with the top 5 dairy counties in NY and the counties with clinical cases of EHD in cattle.

**Figure 3:**
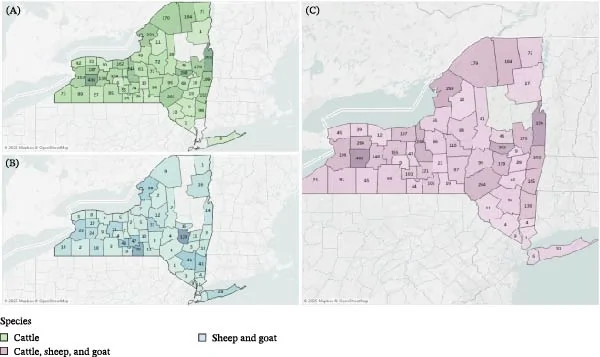
Total number of samples tested over three seasons (July 2022–January 2025). (A) Total number of cattle serum samples tested in New York State by county, July 2022 through January 2025. (B) Total number of sheep and goat serum samples tested in New York State by county, July 2022 through January 2025. (C) Total number of cattle, sheep, and goat serum samples tested in New York State by county, July 2022 through January 2025.

**Table 2:** Number of serum samples by New York State county tested for epizootic hemorrhagic disease virus neutralizing antibody (NA) cumulatively by month for July, August, September, October, November, December, and January for all three seasons.

County	July	August	September	October	November	December	January	Grand total
Albany	2	9	10	1	7	—	—	29
Allegany	19	10	10	5	1	—	—	45
Broome	5	1	2	5	—	6	—	19
Cattaraugus	43	7	7	20	—	4	10	91
Cayuga^a^	36	48	31	48	41	30	12	246
Chautauqua	37	11	12	10	13	—	8	91
Chemung	26	2	7	3	2	1	3	44
Chenango	47	21 (1)	10	5	1	—	2	87
Clinton	26	3	2	31	—	—	10	72
Columbia^b^	42	68 (1)	6	1	40	—	7	165
Cortland	6	2	2	4	2	—	7	23
Delaware	157	40	—	2	2	—	3	204
Dutchess^b^	35	59 (5)	30 (1)	3	—	1	5	139
Erie	90 (1)	14	71	10	6	2	3	197
Essex	1	2	—	—	—	—	14	17
Franklin	72	11	24	61	6	10	—	184
Fulton	8	31	4	3	—	—	—	46
Genesee^a^	26	98	35	34	1	12	—	206
Greene^b^	17	1 (1)	—	2	1	—	—	22
Herkimer	7	4	21	2	6	—	1	41
Jefferson^b^	41	60	24	33	41	11	49	259
Lewis	5	—	4	1	3	—	—	13
Livingston	35	8	58	3	41	—	3	148
Madison	44 (1)	28	8	6	13	10	—	110
Monroe	9	2	—	—	1	—	—	12
Montgomery^c^	35 (1)	106 (2)	55	34	30 (1)	1	38	303
Nassau	—	2	2	1	—	—	1	6
Niagara	34	2	2	—	1	4	2	45
Oneida	60	9	4	8	3	—	—	84
Onondaga	14	38	1	12	1	—	—	66
Ontario	50	65	10	9	5	11	5	155
Orange	1	1	—	—	2	—	—	4
Orleans	26	—	5	1	—	7	—	39
Oswego^b^	1	46 (4)	5	—	—	—	—	56
Otsego	54	29	7	5	2	1	1	99
Putnam	—	4	—	—	—	—	—	4
Rensselaer	76	75	9 (1)	39	1	—	1	202
Rockland	6	—	—	3	—	—	—	9
Saint Lawrence^a^	106	47	2	12	—	11	1	179
Saratoga	53 (1)	9	55 (7)	4	43	3	1	176
Schenectady	4	5	—	—	—	—	—	9
Schoharie	38	19	1	10	110	1	1	180
Schuyler	8	3	4	34	21 (2)	30	1	103
Seneca	8	33	—	2	—	—	4	47
Steuben	22	2	1	—	61	2	—	88
Suffolk^bc^	—	5	1	9	11	5	—	31
Sullivan	2	9	—	—	—	—	—	11
Tioga	47	3	16	16	—	1	22	105
Tompkins	12	36	37	9	6	3	18	121
Ulster^b^	20 (1)	5	7	8	1	2	6	50
Washington^ac^	185 (1)	114 (1)	47	18	5	7	1	379
Wayne	70	4	29	35	4	16	9	167
Westchester	1	—	—	—	—	—	—	1
Wyoming^a^	69 (1)	31	134	1	16	205	3	460
Yates	3	—	2	1	2	—	1	9

Grand total	1841 (7)	1242 (15)	814 (9)	564	553 (3)	397	253	5664 (34)

*Note:* Seropositives are in parenthesis.

^a^Indicates the top five dairy counties in New York State, ranging from 49,000 to 29,500 dairy cattle.

^b^Indicates a county with >50–500 white‐tailed deer deaths attributed to EHD in 2021.

^c^Indicates counties that had EHD clinical cases in cattle.

Cattle breeds included Hereford, Ayrshire, Jersey, Shorthorn, Mini Hereford, Guernsey, Angus, Black Baldy, White Park, Holstein, Polled Hereford, and mixed breed, and sheep breeds included Icelandic and Katahdin.

## 4. Discussion

EHDV is an important vector‐borne virus because it causes hemorrhagic disease and high mortality amongst naïve WTD populations. It also causes disease in mule deer, pronghorn deer, big‐horn sheep, and North American elk [4]. It produces oral vesicular lesions and lameness in cattle and bison, thus mimicking FMD, and cattle are a potential reservoir for EHDV [27]. A large EHD outbreak occurred in the wild WTD population in NY in the summers of 2020 and 2021 [24], coinciding with a small number of EHD cases in domestic cattle and a captive bison herd in 2021. In 2022, roughly 150 WTD deaths were attributed to EHDV in the Hudson Valley from August to October. There were zero cases in 2023 and nine cases in the Hudson Valley in August 2024. This pattern matches trends observed previously in northern US, an incursive zone, where EHDV is relatively rare. This is the opposite of “enzootic stability” observed in parts of the south and southeastern US, where there is high seroprevalence and little mortality due to regular EHDV circulation and the resultant immunity in the WTD population [3, 11, 26, 35].

In 2021, the Cornell Wildlife Health Laboratory created and maintained a NY state map of the number of WTD mortalities attributed to EHDV by county [24]. The following counties had an estimate of >50–500 deaths: Columbia, Dutchess, Greene, Ulster (Hudson Valley region), Jefferson, Oswego (Lake Ontario region), and Suffolk County (Long Island). We tested 165, 139, 22, 50, 259, 56, and 31 serum samples from each county, respectively (Table 2), as part of the passive surveillance utilizing convenience samples received at the AHDC for screening tests. In season 1, 7 of the 10 seropositive samples were from those counties (Columbia, Dutchess, and Oswego), in season 2, 5 of the 15 seropositive samples were from Dutchess County, and in season 3, 1 of the 9 seropositive samples was from Greene County. These results indicate *Culicoides* activity and the presence of EHDV, as we have proof of both clinical cases of EHD in WTD and seroconversion in domestic ruminants without clinical signs. The first EHD outbreak in cattle with clinical disease occurred in Suffolk County in 2021, but we found no seropositive samples in our 3 bovine and 31 small ruminant serum samples from that county tested as part of the passive surveillance for the study. There are very few domestic ruminants in Suffolk County, as it is on Long Island, and the lack of seroprevalence in healthy animals is likely influenced by the small sample size. Moreover, the design of the study precluded sampling of entire herds from EHD “hot spots,” as the sampling was based on convenience samples submitted from the entire state, as opposed to focusing on regions with EHD clinical cases in WTD or cattle. This must be taken into consideration when assessing the low overall seroprevalence as the sample dilution effect is a potential important factor that could contribute to a perceived lower seroprevalence.

Montgomery County had a clinical case of EDHV‐2 in a cow in 2023. There were two seropositive cows in both seasons 1 and 2; the cattle from season 1 were antibody positive for EHDV‐6, and the cattle from season 2 were EHDV‐2. Washington County is on the state border with VT, near the bovine clinical cases in 2021, and had a clinical case of EHDV‐2 in a cow in 2024. There was one EHDV‐2 seropositive bovine sample in both seasons 1 and 2 from Washington County, which is the fourth largest dairy county in NY. The five most populous dairy counties are Cayuga (49,000 cows), Wyoming (48,500), Saint Lawrence (34,500), Washington (30,500), and Genesee County (29,500) [31]. We tested 246, 460, 179, 379, and 206 samples from each county, respectively. There was one seropositive cow in Wyoming (Table 2). Wyoming County is not in the region where EHD mortality occurred in WTD but does have large numbers of cattle. These results could be due to animal movement, *Culicoides* activity, or undetected morbidity and mortality in the WTD. Samples were geographically comprehensive, representing 55 of the 62 counties in NY. There were no samples coming from the five counties in NY City, a major metropolitan area without animal agriculture, leaving just Hamilton and Warren Counties unrepresented, though they have negligible dairy cattle [31]. Although sheep only comprised 4.2% of the samples tested (239/5698), there were three positive ovine samples and no positive goat samples out of 590, though they represented 10.4% of the total samples tested.

There were 3928/5698 samples collected in summer months; 1848 in July, 1,257 in August, and 823 in September (Figure 2), representing 68.9% of the samples. There were 1770/5698 samples collected in the fall and winter; 564 in October, 556 in November, 397 in December, and 253 in January (Figure 2), representing 31.1% of the samples. Thirty‐one of the 34 EHDV seropositive samples were collected during the summer months, with the three other positive samples collected in November (Figure 2), indicating that seroprevalence for EHDV is highest in the summer in NY The half‐life of EHDV antibodies is unknown in domestic ruminants, but BTV‐8 antibodies persisted for up to 6 years in cattle in Germany [36] and EHDV antibodies are generally thought to “persist” for prolonged periods in exposed animals [37]. In this data set, NA could be derived from exposure from previous vector seasons, particularly the 7 July seropositive animals, since historically, the earliest clinical cases of EHD in both cattle and WTD in NY occurred in August. July also had the highest number of samples tested, which could skew data towards a seroprevalence for the previous season. The three seropositive animals in November could be due to exposure during the summer months. One animal that tested positive in August 2022 was only 3 months old, which could have been due to maternal antibodies, as previously discussed, though young animals can test positive for EHDV [27]. Ages of the seropositive animals in the data set ranged from 3 months to 12 years, with most of the animals between 1 and 3 years, reflecting *Culicoides* activity in the recent past. There was no specific pattern to the age of the seropositive animals in the data set.

The northward expansion of EHDV in the US has been correlated with dry, hot summers [17, 18]. The development of *Culicoides* larvae occurs in wet environments, often in association with livestock [10, 15, 38]. *Culicoides* spp. as a vector for EHDV are poorly understood, particularly when compared to BTV [13, 15]. In 1983, Schmidtmann et al. [38] found *Culicoides variipennis* larvae on 46 of 56 NY dairy farms sampled, and no further *Culicoides* research in NY has been published. *Culicoides* collection data from Ontario, Canada, demonstrated peak activity in June [13, 39]. Ontario and NY share a border and present similar environmental conditions, but the WTD mortality events and the clinical cases in cattle and bison occurred in late summer and fall (August through November), months after peak activity in Ontario, Canada. *Culicoides* spp. surveillance in NY, including species composition, habitat and host associations, geographic location, phenology, and viral testing, is needed to understand the vector’s role in EHD during and between outbreaks. Changes in environmental conditions and season temperatures may impact outbreaks of EHD in wild and domestic ruminants due to northern expansion of the range and length of the season of *Culicoides*. The bison from the October 2021 case was real‐time RT‐PCR positive in whole blood 5 months after the initial diagnosis, which was March 2022, with a robust Ct value (28.85). The persistence of viremia could provide evidence for overwintering of the virus, thus maintaining it in the ruminant population for the next *Culicoides* season.

While the EHDV AGID test detects antibodies to EHDV‐1–8, the serum neutralization assays were only performed for EHDV‐1, 2, and 6. Based on the results in Table 1, all cases had NAs to at least one serotype, but it is still possible that some of these results were cross‐reaction to other serotypes that were not tested, including known and unknown serotypes.

The study used convenience samples received at the AHDC and screening tests for EHDV antibodies to provide an initial assessment of the seroprevalence in domestic ruminants across the state of NY. The study is inherently prone to sampling bias and clustering within herds or flocks, and this must be considered when analyzing the outcomes of this study. There were three instances of multiple seropositive animals on the same premise; in season 2, there were five cattle from the same premise in Dutchess County and two sheep from the same premise in Schuyler County, and in season 3, there were seven cattle from the same premise in Saratoga County. Therefore, 41.2% (14/34) of the seropositive samples were potential results of clustering.

International data reveals varying but generally higher, percentages of seropositive animals, particularly in regions where there have been EHD outbreaks. A serosurvey of domestic ruminants in Pakistan from 2019 to 2021 showed 66.9% positivity in cattle, 21.3% in bison, and 4.1% in goats. EHDV‐6 was the most common, but EHDV‐1, 5, and 7 were also found [40]. In China, serum from 18,122 cattle, yaks, small ruminants, and deer was collected from 15 provinces from 2014 to 2019. Overall, 30.1% of the samples were positive, with cattle being more positive than other species, and the highest rate of seropositive animals occurred in the fall [41]. From 2014 to 2017, 2886 serum samples from beef cattle in southern China were tested for EHDV antibodies; the seroprevalence was 57.9%, and EHDV‐1 and 5–8 were present [42]. EHDV‐8 spread from Tunisia in 2021 to southern Europe, where it was detected in Italy, Spain, Portugal, and France in 2022–2023 [37, 43, 44]. In southwestern Spain in 2022, two red deer were diagnosed with EHDV‐8, and a subsequent survey in that region found 6.3% (37–592) of the deer had EHDV antibodies [43]. A seroprevalence study comparing cattle from the affected region (southern France) with the unaffected region (northern France) was performed. A total of 2763 serum samples showed that in the south, 82.6% of cattle had EHDV antibodies, and in the north, 11.6% had antibodies [37].

Only a few recent EHDV prevalence studies were conducted in the US in cattle and bison, with none in the Northeast. The results varied greatly, like studies in Asia and Europe. In 2023, Krus et al. [30] tested 287 North American bison from seven Western and Midwestern US states and found 57.5% EHDV seroprevalence. Merrill et al. [27] performed PCR testing on 1604 beef cattle in Florida from 2015 to 2017, and 15 animals (0.9%) were positive for EHDV. In 2014, Hairgrove et al. [45] tested 308 auction cattle in Texas and found 97.8% seroprevalence. Boyer et al. [29] surveyed 9414 bovine serum samples from 60 herds over 3 years (2000, 2001, and 2002) in Illinois and Indiana, and found a seroprevalence of 11.8%. McVey et al. [46] performed ELISA testing for *Orbiviruse* on cattle and various wild deer species in 2016 and found a seroprevalence of 51% in Kansas and 25% in Nebraska. WTD serology studies are even more scarce. In 1998, 2001, and 2002, EHDV serological testing from 87 WTD in Kansas revealed 67% seroprevalence [47]. In 1991 and 1992, EHDV serology in Texas was performed on 574 WTD, and 84% were seropositive [26], but this data is almost 30 years old.

When compared to international and national prevalence studies of EHD in wild and domestic populations, our data set detected 0.6% seroprevalence of EHDV in 5698 cattle, sheep, and goats in NY over three seasons, which was a very low but nonnegligible level of exposure and consequently, immunity to the virus. This may be attributed to the cyclical nature of the virus in the Northeastern US compared to other regions of the world, the spread of vectors and susceptible populations, and the proximity of WTD to cattle, depending on the geography and animal density. This data provides a broad foundation for longitudinal and geographically localized testing for EHD (for example, Montgomery and Washington Counties, which had both EHD clinical cases and seropositive animals) within the northeastern US to understand EHD dynamics on the landscape, serotype prevalence, and persistence of the virus in the northeastern US.

## 5. Conclusion

These findings provide critical information to several stakeholders, including conservation biologists, veterinarians, cattle producers, and state and federal regulatory officials, on the epidemiology of EHDV. Our data combine clinical outbreaks in cattle and bison with seroprevalence in cattle, sheep, and goats during and after an EHDV outbreak in WTD in a region where EHDV circulation is not endemic.

## Funding

This research was supported by the intramural research program of the United States Department of Agriculture, National Institute of Food and Agriculture, Federal Capacity Funds (Hatch Grant 7003411).

## Disclosure

The findings and conclusions in this preliminary publication have not been formally disseminated by the U.S. Department of Agriculture and should not be construed to represent any agency determination or policy.

## Ethics Statement

This study did not involve the use of live animals; therefore, ethical approval was not required. All serum samples were submitted by licensed veterinarians for other routine diagnostic testing; they were not solicited for this research study. Samples become property of the Cornell University Animal Health Diagnostic Center upon receipt.

## Conflicts of Interest

The authors declare no conflicts of interest.

## Data Availability

The data that support the findings of this study are available from the corresponding author upon reasonable request.
